# Online Least Squares One-Class Support Vector Machines-Based Abnormal Visual Event Detection

**DOI:** 10.3390/s131217130

**Published:** 2013-12-12

**Authors:** Tian Wang, Jie Chen, Yi Zhou, Hichem Snoussi

**Affiliations:** 1 Institut Charles Delaunay-LM2S-UMR STMR 6279 CNRS, University of Technology of Troyes, Troyes 10004, France; E-Mail: hichem.snoussi@utt.fr; 2 Observatoire de la Côte d'Azur-UMR 7293 CNRS, University of Nice Sophia-Antipolis, Nice 06108, France; E-Mail: jie.chen@unice.fr; 3 College of Information Science and Technology, Dalian Maritime University, Dalian 116026, China; E-Mail: yi.zhou@dlmu.edu.cn

**Keywords:** abnormal detection, optical flow, covariance matrix descriptor, online least squares one-class SVM

## Abstract

The abnormal event detection problem is an important subject in real-time video surveillance. In this paper, we propose a novel online one-class classification algorithm, online least squares one-class support vector machine (online LS-OC-SVM), combined with its sparsified version (sparse online LS-OC-SVM). LS-OC-SVM extracts a hyperplane as an optimal description of training objects in a regularized least squares sense. The online LS-OC-SVM learns a training set with a limited number of samples to provide a basic normal model, then updates the model through remaining data. In the sparse online scheme, the model complexity is controlled by the coherence criterion. The online LS-OC-SVM is adopted to handle the abnormal event detection problem. Each frame of the video is characterized by the covariance matrix descriptor encoding the moving information, then is classified into a normal or an abnormal frame. Experiments are conducted, on a two-dimensional synthetic distribution dataset and a benchmark video surveillance dataset, to demonstrate the promising results of the proposed online LS-OC-SVM method.

## 1. Introduction

**Figure 1.**
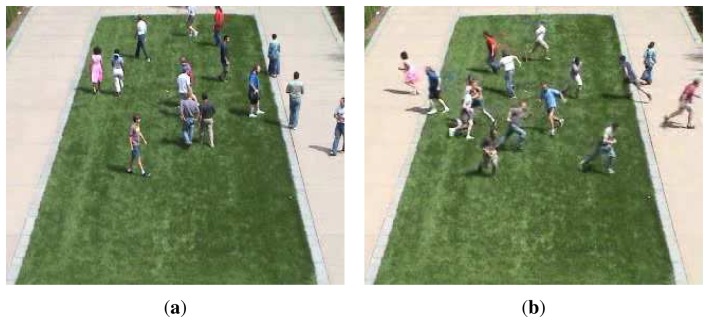
Examples of the normal and abnormal scenes. (**a**) A normal lawn scene: all the people are walking; (**b**) An abnormal lawn scene: all the people are running.

Visual surveillance is one of the major research areas in computer vision. After recording events by a visual sensor, such as a camera, obtaining detailed information of individual or crowd behavior is a challenging object in this area; automatic abnormal event detection is required to provide convenience, safety and an efficient lifestyle for humanity [1]. An abnormal event is defined as behavior deviating from what one expects. For example, a pedestrian panic in a public region: the people are running in the plaza, where people are usually strolling. As shown in Figure 1a, a normal scene is illustrated, the people are walking. In Figure 1b, the people are suddenly running in different directions; this scene is considered abnormal. If a system can detect this event, which might imply a safety risk, security staff can be alerted to take emergency response procedures. The abnormal event detection is a content-based video analysis problem; it includes two technologies: a feature representation of an event model and an abnormal event detection approach.

In [2–4], abnormal detection approaches with behavioral models were introduced. The behavior pattern modeled by adopting optical flow or pixel change history (PCH) was represented by Bayesian models. In [5], the motion feature, including the position, direction and velocity, was modeled by latent Dirichlet allocation. In [6], the abnormal vehicle behavior at intersections was detected via a stochastic graph model based on the Markovian approach. The behavior was labeled as abnormal when the current motion pattern cannot be recognized as any state of the system or a particular sequence of states cannot be parsed with the stochastic model. These works relied on an explicit signal statistical model, and the abnormal events were the ones interpreted as statistical model abrupt changes, maximum likelihood or Bayesian estimation theory [7]. The signal model together with probabilistic assumption techniques are usually extremely powerful insofar as an accurate model ex0ists; these methods are effective in several scenarios. However, there are various situations where a robust and tractable model cannot be obtained. This raises the need for model-free methods.

On the other hand, low-level motion features were employed. In [8], the authors presented an algorithm monitoring optical flow in a set of fixed spatial positions. The similarity of the model was computed to detect abnormal patches. In [9], the irregular behavior of images and videos was detected by comparing the likelihood of patches via a probabilistic graphical model. These methods based on separated patches, benefiting from the partial knowledge of the image, do not exploit the global information of the frame.

Trajectory information is also adopted to detect abnormal events. In [10,11], the authors presented a method for anomalous event detection by means of trajectory analysis. The trajectories were subsampled to a fixed-dimension vector representation and clustered with an one-class support vector machine (SVM). In [12], alarm detection of traffic was performed on the basis of the parameters of the moving objects and their trajectories by using semantic reasoning and ontologies. In [13], vision-based abnormal events for home healthcare systems were detected by using shape feature variation and 3D trajectory. Tracking-based algorithms are likely to fail in crowded scenes.

We consider the model-free approach, which does not require an explicit statistical model. To be accurate, the support vector machine (SVM) classification method is relied on in this paper. Inspired by the satisfactory performance of a covariance feature descriptor representing object in a tracking problem, a covariance descriptor characterizes the moving information of a global frame. In a tracking problem, the covariance descriptor is constructed of the blob intensity or color for template matching. In this paper, covariance encodes the optical flow of the global frame.

The rest of the paper is organized as follows. In Section 2, related works are briefly reviewed. In Section 3, the online least squares one-class support vector machine (online LS-OC-SVM) classification method is originally derived. In Section 4, a covariance matrix descriptor is described to provide feature vectors for the classification algorithm. In Section 5, we propose abnormal detection methods based on the online LS-OC-SVM. In Section 6, we present the results on synthetic data and real-world video scenes. Finally, Section 7 concludes the paper.

## 2. Related Work

SVM is usually trained in a batch model, *i.e.*, all training data are given *a priori* and are learned together. If additional training data arrive afterward, the SVM must be retrained from scratch [14]. In the problem of abnormal event detection in video surveillance, the normal sequence for training may last for a long time. It is impractical to train the big training set of normal samples as one batch together. If a new datum is added to a large training set, it will likely have only a minimal effect on the previous decision surface. Resolving the problem from scratch seems computationally wasteful. Considering these two aspects, the online strategy is considered in our work to adapt to the computational and the memory requirement.

Some online learning algorithms for SVM were derived based on analyzing the change of Karush-Kuhn-Tucker (KKT) conditions while updating the classifier. In [15], new arrival data along with the data violating the KKT conditions, and the support vectors from the last iteration, were considered as a new training dataset to train the classifier at the current step. The iteration will be stopped when all data satisfy the KKT conditions. In [16,17], the authors analyzed the change of the KKT conditions when one datum was included into, or removed from, the training set; then, a so-called bookkeeping step was used to compute the new coefficients of the classifier to achieve an online update for a two-class SVM. Useful implementation issues on incremental SVM were presented in [18].

In [7], it was argued that the binary classification algorithm in [16] cannot be directly implemented for a one-class problem. In [7,19], the authors considered the change of the normal model over time and online identified outliers using previous data vectors in a sliding time window. Two one-class SVM classifiers, which preceded and followed the present instant, were compared. A change in the statistics of the time series was likely to occur when the resulting machines were different. The sliding time window approach was considered in [20], with an application on wireless sensor networks. This method, adopting sliding window formulation, is not inherently online, since it requires repeated batch training of new machines.

In [21], an online one-class SVM was presented following the idea of [22]: an exponential window was applied to the data to suit it to an adaptive scenario where the solution was able to track the changes of the data distribution and to forget old patterns. This algorithm is based on the slow-varying assumption.

Some online one-class SVM classification methods were proposed based on support vector data description (SVDD) [23,24], the hypersphere one-class SVM formulation. In [25], an online one-class classification method was proposed, a least squares optimization problem was considered and the model complexity was controlled by the coherence criterion. In [26], a method was proposed to reduce space and time complexities. It reduced the training set size during the training procedure by removing data having a high probability of becoming non-support-vectors.

In order to sidestep the difficulty in the nature of the constrained quadratic optimization problem, we derive an online version of the hyperplane one-class SVM [27] based on the least squares regularization. In the least squares SVM version, one finds the solution by solving a linear system instead of a quadratic programming problem. This advantage comes from the use of equality instead of inequality constraints in the problem formulation [28]. Least squares one-class SVM (LS-OC-SVM) was proposed in [29], without considering the sparsity of the hyperplane representation. It is thus inappropriate to detect abnormal events online. In the following, we shall derive an online version of the least squares one-class SVM, then propose a sparsification representation of the detector.

## 3. Classification

In this section, we introduce the derivation of the proposed online least square one-class support vector machine (online LS-OC-SVM). In abnormal detection problems, it is supposed that the samples from a positive class are obtainable. A density will only exist if the underlying probability measure possesses an absolutely continuous distribution function, but the general problem of estimating the measure for a large class of sets is not solvable [27,30]. The one-class SVM framework is then suitable to the specificity of the abnormal event detection where only normal scene data are available. Support vector machine (SVM) was initially proposed by Vapnik and Lerner [31], attempting to find a compromise between the minimization of empirical risk and the prevention of overfitting. By applying a kernel trick, SVM can handle nonlinear classification problems [10,32–34]. Based on the theoretical foundation of SVM and the soft-margin trick [35,36], one-class SVM is proposed to address the problem where only one-category (the positive) samples with a few outliers are available. In this section, after a brief review of one-class SVM and least squares one-class SVM on a batch model, an online training algorithm is proposed. A sparsified version of the algorithm will then be provided for further adapting to critical online requirements.

### 3.1. One-Class SVM

One-class SVM (OC-SVM) aims to determine a suitable region in the input data space, *χ*, which includes most of the samples drawn from an unknown probability distribution, *P*. It detects objects that resemble training samples. The hypersphere one-class SVM was proposed in [23,24]. It identified outliers by fitting a hypersphere with a minimal radius. The hyperplane one-class SVM was an extended version of the original SVM to one-class problems [27,36]. It identified outliers by fitting a hyperplane from the origin. In our work, we adopt the hyperplane one-class SVM, which is formulated as a constrained minimization optimization problem:

(1) $$\underset{\omega ,\xi ,\rho }{\min}\frac{1}{2}∥w{∥}^{2}-\rho +C\sum_{i=1}^{n}\xi_{i}\;\text{subject to}\;\langle w,\Phi \left({\text{x}}_{i}\right)\rangle \geq \rho -\xi_{i},\;\xi_{i}\geq 0$$

where *x_i_* ∈ *χ*, *i* ∈ {1…*n*}, are *n* training samples in the input data space, *χ*, and *ξ_i_* is the slack variable for penalizing the outliers. The hyperparameter, *C*, is the weight for restraining the slack variable. It tunes the number of acceptable outliers and, thus, enables the analyzing of noisy data points. ‖·‖ denotes the Euclidean norm of a vector. The decision hyperplane is given by the equation:

(2) $$\langle w,\Phi ({\text{x}}_{i})\rangle -\rho =0$$

The nonlinear function, Φ : *χ* **→ 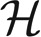
**, maps datum ***x****_i_* from the input space, *χ*, into the feature space, ***
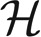
***, which allows us to solve a nonlinear classification problem by designing a linear classifier in the feature space. *w* defines a hyperplane in the feature space separating the projections of training data from the origin. A positive definite kernel function, *κ*, is defined as *κ*(***x***, ***x′***) = <Φ(***x***), Φ(***x′***)〉, which implicitly maps the training or testing data, ***x***, into a higher (possibly infinite) dimensional feature space. Introducing the Lagrangian multipliers, α*_i_*, the decision function in the input data space, *χ*, is given by:

(3) $$f(\text{x})=\mathrm{sgn}(\sum_{i=1}^{n}\alpha_{i}\kappa ({\text{x}}_{i},\text{x})-\rho )$$

if *f*(***x***) = −1, the datum, ***x***, is classified as abnormal; otherwise, ***x*** is classified as normal.

### 3.2. Least Squares One-Class SVM

Least squares SVM (LS-SVM) was proposed by Suykens in [37,38]. By using the quadratic loss function, Choi proposed least squares one-class SVM (LS-OC-SVM) [29]. LS-OC-SVM extracts a hyperplane as an optimal description of training objects in a regularized least squares sense. It can be written as the following objective function:

(4) $$\underset{\omega ,\xi ,\rho }{\min}\frac{1}{2}∥\text{w}{∥}^{2}-\rho +\frac{1}{2}C\sum_{i=1}^{n}\xi_{i}^{2}\;\text{subject to}\;\langle \text{w},\Phi \left({\text{x}}_{i}\right)\rangle =\rho -\xi_{i}$$

The condition for the slack variables in OC-SVM, *ξ_i_* ≥ 0, is no longer in need. The variable, *ξ_i_*, represents an error caused by a training object, ***x****_i_*, with respect to the hyperplane. The definitions of the other parameters in Equation (4) are the same as the ones in OC-SVM. The associated Lagrange is:

(5) $$L=\frac{1}{2}∥\text{w}{∥}^{2}-\rho +\frac{C}{2}\sum_{i=1}^{n}\xi_{i}^{2}-\sum_{i=1}^{n}\alpha_{i}\;\left({\text{w}}^{⊤}\Phi \left({\text{x}}_{i}\right)-\rho +\xi_{i}\right)$$

Setting derivatives of Equation (5) with respective to primal variables, ***w***, *ξ_i_*, *ρ* and *α_i_*, to zero, we have the following stationarity conditions:

(6) $$\begin{array}{cc} \frac{\partial L}{\partial \text{w}}=0 & \Rightarrow \text{w}=\overset{n}{\underset{i=1}{\text{∑}}}\alpha_{i}\Phi \left({\text{x}}_{i}\right) \end{array}$$

(7) $$\begin{array}{cc} \frac{\partial L}{\partial \xi_{i}}=0 & \Rightarrow C\xi_{i}=\alpha_{i} \end{array}$$

(8) $$\begin{array}{cc} \frac{\partial L}{\partial \rho }=0 & \Rightarrow \overset{n}{\underset{i=1}{\text{∑}}}\alpha_{i}=1 \end{array}$$

(9) $$\begin{array}{cc} \frac{\partial L}{\partial \alpha_{i}}=0 & \Rightarrow {\text{w}}^{⊤}\Phi \left({\text{x}}_{i}\right)+\xi_{i}-\rho =0 \end{array}$$

Substituting Equations (6)–(8) into (9) yields:

(10) $$\sum_{i,j=1}^{n}\alpha_{i}\Phi^{⊤}({\text{x}}_{i})\Phi ({\text{x}}_{j})+\frac{{\text{α}}_{i}}{C}-\rho =0$$

For all *I* = 1, 2,…, *n*, we can rewrite Equation (10) in matrix form as:

(11) $$\left[\begin{array}{cc} \text{K}+\frac{\text{I}}{C} & 1\\ 1^{⊤} & 0 \end{array}\right]\;\left[\begin{array}{c} \text{α}\\ -\rho \end{array}\right]=\left[\begin{array}{c} 0\\ 1 \end{array}\right]$$

where ***K*** is the Gram matrix with (*i*, *j*)-th entry *κ*(***x****_i_*, ***x****_j_*), ***I*** is the identity matrix with the same dimension as Gram matrix ***K*** and ***α*** is the column vector with *i*-th entry *α_i_* for training sample ***x****_i_*. 1 and 0 are all-one and all-zero column vectors, respectively, with compatible lengths. The parameters, ***α*** and *ρ*, could be obtained by:

(12) $$\left[\begin{array}{c} \text{α}\\ -\rho \end{array}\right]={\left[\begin{array}{cc} \text{K}+\frac{\text{I}}{C} & 1\\ 1^{⊤} & 0 \end{array}\right]}^{-1}\left[\begin{array}{c} 0\\ 1 \end{array}\right]$$

The hyperplane is then described by:

(13) $$f\left(\text{x}\right)=\sum_{i=1}^{n}{\text{α}}_{i}\kappa \left({\text{x}}_{i},\text{x}\right)-\rho =0$$

The distance, *dis*(***x***), of a datum, ***x***, with respect to the hyperplane is calculated by:

(14) $$\text{dis}(\text{x})=\frac{\left|f(\text{x})\right|}{\left\|\text{α}\right\|}=\frac{\left|\left(\sum_{i=1}^{n}\alpha_{i}\kappa ({\text{x}}_{i},\text{x})-\rho \right)\right|}{\left\|\text{α}\right\|}$$

where ***x****_i_* is a training sample, ‖***α***‖ is the two-norm of vector ***α***. An object with a low *dis*(***x***) value lies close to the hyperplane thus resembles the training set better than other objects with high *dix*(***x***) values. The distance, *dis*(***x***), is used as a proximity measure to determine the normal and abnormal class of the data [29].

### 3.3. Online Least Squares One-Class SVM

In an online learning scheme, the training data continuously arrive. We thus need to tune hyperparameters in the objective function and the hypothesis class in an online manner [17]. Let ***α**_n_*, ***K****_n_* and ***I****_n_* denote the coefficient, Gram matrix and identity matrix at the time step, *n*, respectively. The parameters of LS-OC-SVM [***α****_n_* – *ρ_n_* ]^T^ at the time step, *n*, could be calculated as:

(15) $$\left[\begin{array}{c} {\text{α}}_{n}\\ -\rho_{n} \end{array}\right]={\left[\begin{array}{cc} {\text{K}}_{n}+\frac{{\text{I}}_{n}}{C} & 1_{n}\\ 1_{n}^{⊤} & 0 \end{array}\right]}^{-1}\left[\begin{array}{c} 0_{n}\\ 1 \end{array}\right]$$

In order to proceed, recall the matrix inverse identity for matrices *A*, *B*, *C* and *D* with suitable sizes [39]:

(16) $${\left[\begin{array}{cc} A & B\\ C & D \end{array}\right]}^{-1}=\left[\begin{array}{cc} A^{-1} & 0\\ 0 & 0 \end{array}\right]+\left[\begin{array}{c} \begin{array}{c} -A^{-1}B \end{array}\\ 1 \end{array}\right]\times {\left(D-CA^{-1}B\right)}^{-1}\times [\begin{array}{cc} -CA^{-1} & 1 \end{array}]$$

The matrix, ***K****_n_*, with diagonal loading $\frac{{\text{I}}_{n}}{C}$ can be calculated recursively with respect to time step *n* by:

(17) $${\left[{\text{K}}_{n+1}+\frac{{\text{I}}_{n+1}}{C}\right]}^{-1}$$

(18) $$={\left[\begin{array}{cc} {\text{K}}_{n}+\frac{\text{I}}{C} & {\text{κ}}_{n+1}\\ {\text{κ}}_{n+1} & \kappa_{n+1}+\frac{1}{C} \end{array}\right]}^{-1}$$

(19) $$\begin{array}{cccc} =\left[\begin{array}{cc} {\left({\text{K}}_{n}+\frac{{\text{I}}_{n}}{C}\right)}^{-1} & 0_{n}\\ 0_{n}^{⊤} & 0 \end{array}\right]+ & \frac{1}{\left(\kappa_{n+1}+\frac{1}{C}\right)-\kappa_{n+1}{\left({\text{K}}_{n}+\frac{{\text{I}}_{n}}{C}\right)}^{-1}{\text{κ}}_{n+1}} & \left[\begin{array}{c} \begin{array}{cc} -{\left({\text{K}}_{n}+\frac{{\text{I}}_{n}}{C}\right)}^{-1} & {\text{κ}}_{n+1} \end{array}\\ 1 \end{array}\right] & \left[-{\text{κ}}_{n+1}^{⊤}{\left({\text{K}}_{n}+\frac{{\text{I}}_{n}}{C}\right)}^{-1}1\right] \end{array}$$

where ***κ****_n_*_+1_ is the column vector with *i*-th entry ***κ***(***x****_i_*, ***x****_n_*_+1_), *i* ∈ {1, 2, …, *n*}, and *κ_n_*_+1_ = *κ*(***x****_n_*_+1_, ***x****_n_*_+1_). Based on Equations (15) and (17), we arrive at an online implementation of LS-OC-SVM.

### 3.4. Sparse Online Least Squares One-Class SVM

The procedures for calculating the parameters, ***α*** and *ρ*, of LS-OC-SVM in Section 3.3 lose sparseness, due to the quadratic loss function in the objective function Equation (4). This formulation is inappropriate for large-scale data and unsuitable for online learning, as the number of training samples grows infinitely [25]. We propose a sparse solution to provide a robust formulation. A dictionary is adopted to address the sparse approximation problem [40].

Instead of Equation (6), where ***w*** is expressed with all available data, we intend to approximate it by adopting a dictionary in a sparse way. Consider a dictionary, ***x***_
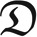
_, 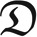 ⊂ {l, 2, …, *n*}, of size *D* with elements ***x****_w_j__*, *j* ∈ 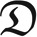. Instead of Equation (6), we approximate ***w*** with these *D* dictionary elements:

(20) $$\text{w}=\sum_{j=1}^{D}\beta_{j}\Phi \left({\text{x}}_{w_{j}}\right)$$

The hyperplane becomes:

(21) $$f(\text{x})=\sum_{j=1}^{D}\beta_{j}\kappa \left(\text{x},{\text{x}}_{w_{j}}\right)-\rho =0$$

In sparse online LS-OC-SVM, the distance, *dis*_
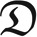
_ (***x***), of a datum, ***x***, to the hyperplane is:

(22) $$dis_{D}(\text{x})=\frac{\left|\sum_{j=1}^{D}\beta_{i}\kappa (\text{x},{\text{x}}_{w_{j}})-\rho \right|}{\left\|\beta \right\|}$$

Where *x_w_j__* is a dictionary element and ***β*** is the column vector with the entries, *β_j_*. Replacing Expression (20) into Lagrange Function (5), we have:

(23) $$L=\frac{1}{2}{\text{β}}^{⊤}K_{D}\text{β}-\rho +\frac{C}{2}\sum_{i=1}^{n}\xi_{i}^{2}-\sum_{i=1}^{n}\alpha_{i}(\sum_{j=1}^{D}\beta_{j}\Phi^{⊤}({\text{x}}_{w_{j}})\Phi ({\text{x}}_{i})+\xi_{i}-\rho )$$

Taking the derivatives of the Function (23) with respect to primal variables, ***β***, *ξ_i_*, *ρ* and *α_i_*, yields:

(24) $$\begin{array}{cc} \frac{\partial L}{\partial \text{β}}=0 & \Rightarrow K_{D}\text{β}=K_{D}^{⊤}(\text{x})\text{α} \end{array}$$

(25) $$\begin{array}{cc} \frac{\partial L}{\partial \xi_{i}}=0 & \Rightarrow C\xi_{i}=\alpha_{i} \end{array}$$

(26) $$\begin{array}{cc} \frac{\partial L}{\partial \rho }=0 & \Rightarrow \end{array}\sum_{i=1}^{n}\alpha_{i}=1$$

(27) $$\begin{array}{cc} \frac{\partial L}{\partial \alpha_{i}}=0 & \Rightarrow \overset{D}{\underset{j=1}{\text{∑}}}\beta_{j}\kappa ({\text{x}}_{w_{j}},{\text{x}}_{i})+\xi_{i}-\rho =0 \end{array}$$

The matrix form for Condition (27) is written:

(28) $${\text{K}}_{D}(\text{x})\text{β}+\text{ξ}-\rho =0$$

Replacing Conditions (24) and (25) into (28) leads to:

(29) $${\text{K}}_{D}(\text{x}){\text{K}}_{D}^{-1}{\text{K}}_{D}^{\text{⊤}}\left(\text{x}\right)\text{α}+\frac{\text{α}}{C}-\rho =0$$

Combining Equations (26) and (29), the equation for computing coefficients [***α*** − *ρ*]^T^ becomes:

(30) $$\left[\begin{array}{cc} K_{D}(\text{x}){\text{K}}_{D}^{-1}{\text{K}}_{D}^{⊤}(\text{x})+\frac{\text{I}}{C} & 1\\ 1^{⊤} & 0 \end{array}\right]\left[\begin{array}{c} \text{α}\\ -\rho \end{array}\right]=\left[\begin{array}{c} 0\\ 1 \end{array}\right]$$

After providing these relations with the dictionary, we now discuss the dictionary construction. The coherence criterion is adopted to characterize a dictionary in sparse approximation problems. It provides an elegant model reduction criterion with a less computationally-demanding procedure [25,40,41]. The coherence of a dictionary is defined as the largest correlation between the elements in the dictionary, *i.e.*,

(31) $$\mu =\underset{i,j\in D,i\neq j}{\max}\left|\kappa ({\text{x}}_{i},{\text{x}}_{j})\right|$$

In the online case, the coherence between a new datum and the current dictionary is calculated by:

(32) $$\epsilon_{t}=\underset{j\in D}{\max}\left|\kappa ({\text{x}}_{t},{\text{x}}_{w_{j}})\right|$$

Where ***x****_w_j__* is the element in the dictionary, ***x***_
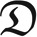
_. Presetting a threshold, *μ*_0_, the new arrival sample, ***x****_t_*, at the time step, *t*, is tested with the coherence criterion to judge whether the dictionary remains unchanged or is incremented by including the new element. For *n* training samples, the subset, which includes *m* (1 ≤ *m* ≪ *n*) samples, is considered the initial dictionary. Then, each remaining sample is tested with Equation (32) to determine the relation between itself and the previous dictionary. If *ϵ_t_* ≤ *μ*_0_, it will be included into the dictionary. Concretely, the algorithm is performed with two cases described herein below.

**First case:***ϵ_t_* > *μ*_0_
  In this case, at time step *n* + 1, the new data, ***x****_n_*_+1_, is not included into the dictionary. The Gram matrix, ***K***_
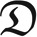
_, with the entries, *κ*(***x****_i_*, ***x****_j_*), *i*, *j* ∈ {1, 2, …, *D*}, is unchanged. When a new sample, ***x***, arrives, we need to compute:
  (33) $$\begin{array}{cc} {\left[\left[\begin{array}{c} {\text{K}}_{D}(\text{x})\\ {\text{κ}}^{⊤} \end{array}\right]{\text{K}}_{D}^{-1}\left[\begin{array}{cc} {\text{K}}_{D}{\left(\text{x}\right)}^{⊤} & \text{κ} \end{array}\right]+\frac{\text{I}}{C}\right]}^{-1}= & {\left[\begin{array}{cc} {\text{K}}_{D}(\text{x}){\text{K}}_{D}^{-1}{\text{K}}_{D}^{⊤}(\text{x})+\frac{\text{I}}{C} & {\text{K}}_{D}(\text{x}){\text{K}}_{D}^{-1}\text{κ}\\ {\text{κ}}^{⊤}{\text{K}}_{D}^{-1}{\text{K}}_{D}^{⊤}(\text{x}) & {\text{κ}}^{⊤}{\text{K}}_{D}^{-1}\text{κ}+\frac{\text{I}}{C} \end{array}\right]}^{-1} \end{array}$$
  where at time step *n* + 1, ***κ*** is the column vector with entries *κ*(***x****_n_*_+1_,***x****_w_j__*), *j* ∈ {1, 2, …, *D*}. ***K***_
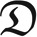
_ (***x***) is the matrix with the (*i*, *j*)-th entry *κ*(***x****_i_*, ***x****_w_j__*), *i* ∈ {1, 2, …, *n*}, *j* ∈ {1, 2, …, *D*}.
**Second case:** *ϵ*_t_ ≤ *μ*_0_
  In this case, the new data, *x_n_*_+1_, is added into the dictionary, ***x***_
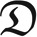
_. Then, the Gram matrix should be changed by:
  (34) $${\bar{\text{K}}}_{D}=\left[\begin{array}{cc} {\text{K}}_{D} & d\\ d^{⊤} & d \end{array}\right]$$

where ***K̅***_
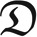
_ is the Gram matrix of the dictionary, including the new arrival dictionary sample, ***x****_n_*_+1_, and ***K***_
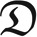
_ is the Gram matrix of the dictionary at the last time step, *n*. Let ***x***_
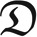
_ = {***x****_w_*_1_, ***x****_w_*_2_, …, ***x****_w_D__* } denote the dictionary at time step *n*; ***d*** is the column vector with entries *d_j_= κ*(***x***,***x****_w_j__*), *j* ∈ {1, 2, …, *D*}, and *d* = *κ*(***x****_n_*_+1_, ***x****_n_*_+1_). By adopting the matrix inverse identity Equation (16), we have:

(35) $${\bar{\text{K}}}_{D}^{-1}=\left[\begin{array}{cc} {\text{K}}_{D}^{-1}+\text{A} & b\\ b^{\text{⊤}} & c \end{array}\right]$$

where:

(36) $$c=\frac{1}{d-{\text{d}}^{⊤}{\text{K}}_{D}^{-1}\text{d}}$$

(37) $$\text{A}=c{\text{K}}_{D}^{-1}\text{d}{\text{d}}^{⊤}{\text{K}}_{D}^{-1}$$

(38) $$\text{b}=-c{\text{K}}_{D}^{-1}\text{d}$$

Because the dictionary changes, the value of ***K***_
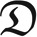
_(***x***) and also ${\left[{\text{K}}_{D}\left(\text{x}\right){\text{K}}_{D}^{-1}{\text{K}}_{D}^{\text{⊤}}\left(\text{x}\right)+\frac{\text{I}}{C}\right]}^{-1}$ should be updated. Let the *S* denote the updated ${\left[{\text{K}}_{D}\left(\text{x}\right){\text{K}}_{D}^{-1}{\text{K}}_{D}^{\text{⊤}}\left(\text{x}\right)+\frac{\text{I}}{C}\right]}^{-1}$ at time step *n* + 1; we have:

(39) $$S={\left[\left[{\text{K}}_{D}\left(\text{x}\right)\text{q}\right]{\bar{\text{K}}}_{D}^{-1}\left[\begin{array}{c} {\text{K}}_{D}^{\text{⊤}}(\text{x})\\ {\text{q}}^{⊤} \end{array}\right]+\frac{\text{I}}{C}\right]}^{-1}$$

(40) $$={\left[{\text{K}}_{D}(\text{x}){\text{K}}_{D}^{-1}{\text{K}}_{D}{\left(\text{x}\right)}^{T}+\frac{\text{I}}{C}+{\text{K}}_{D}(\text{x})\text{A}{\text{K}}_{D}^{⊤}(\text{x})+\text{q}{\text{b}}^{⊤}{\text{K}}_{D}^{\text{⊤}}(\text{x})+{\text{K}}_{D}(\text{x})\text{b}{\text{q}}^{⊤}+c\text{q}{\text{q}}^{⊤}\right]}^{-1}$$

where at time step *n* + 1, ***q*** is the column vector with entries *q_i_* = *κ*(***x****_i_*, ***x****_D_*_+1_), *i* ∈ {1, 2, …, *n*}, and ***x****_D_*_+1_ is the new arrival datum ***x****_n_*_+1_, which is included into the dictionary. The matrix inverse in Equation (39) can be calculated by using four-times Woodbury identity:

(41) $${\left(A+\text{UCV}\right)}^{-1}=A^{-1}-A^{-1}U{\left(C^{-1}+V\;A^{-1}U\right)}^{-1}V\;A^{-1}$$

with proper choices of matrices *A*, *U*, *C* and *V*, such that *U* and *V* should be chosen as two vectors, and *A* should be chosen as a scaler. Thus, the inverse, (*C*^−1^ + *V A*^−1^*U*), is a scaler; Equation (39) can be calculated very efficiently. For instance, for computing the inverse, including the term, (***K***_
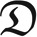
_(***x***)***bq***^⊤^), we regard two vectors, (***K***_
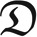
_(***x***)***b***) and ***q***^⊤^, as vector *U* and *V*, respectively, while *C* in Equation (41) is one.

Once knowing *S*, using Equation (33) to add the new ***κ*** with entries *κ*(***x****_n_*_+1_, ***x****_w_j__*), *j* ∈ {1, 2, …,*D*, *D* + 1}, ***x****_w_j__* is an element of the dictionary.

## 4. Covariance Descriptor of Frame Behavior

The optical flow is chosen as the basic low-level feature to represent the movement direction and amplitude. We apply the Horn-Schunck (HS) method to compute optical flow in this paper. The optical flow can provide important information about the spatial arrangement of the object and the change rate of this arrangement [42]. The optical flow of a gray image is formulated as the minimization of the following global energy functional:

(42) $$E=\int \int \left[{\left(I_{x}u+I_{y}\upsilon +I_{t}\right)}^{2}+\gamma^{2}({\left\|\nabla u\right\|}^{2}+{\left\|\nabla \upsilon \right\|}^{2})\right]\text{dxdy}$$

where *I* is the intensity of the image, *I_x_*, *I_y_* and *I_t_* are the derivatives of the image intensity value along the *x*, *y* and time *t* dimension, *u* and *υ* are the components of the optical flow in horizontal and vertical direction and γ represents the weight of the regularization term.

**Figure 2.**
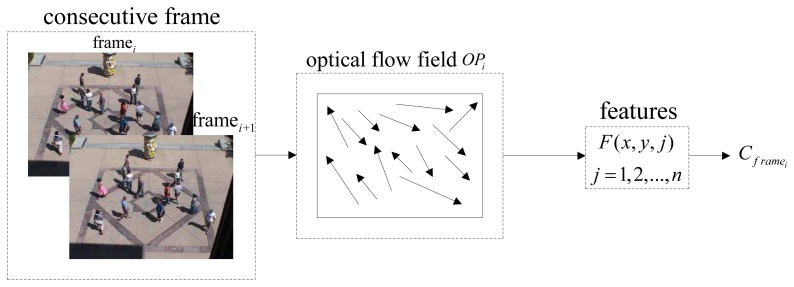
Covariance descriptor computation based on the features of a video frame.

The covariance feature descriptor was originally proposed by Tuzel [43] for pattern matching in a target tracking problem. Owing to its good performance, the covariance descriptor encoding the optical flow is introduced to represent the global movement of the frame. A feature is defined as:

(43) $$F(x,y,i)=\phi_{i}(I,x,y)$$

where *I* is the image (which could be gray, red-green-blue (RGB), hue-saturation-value (HSV), hue-lightness-saturation (HLS), *etc*), *ϕ_i_* is a mapping relating the image with the *i* – *th* feature, *F* is the *W* × *H* × *d* dimension feature, *W* is the image width, *H* is the image height and *d* is the number of the chosen features. For each frame, the feature, *F*, can be represented as the *d* × *d* covariance matrix:

(44) $$C=\frac{1}{n-1}\sum_{k=1}^{n}(z_{k}-\mu ){\left(z_{k}-\mu \right)}^{⊤}$$

where *n* is the number of the pixels sampled in the frame, ***μ*** is the mean of *n* feature vectors of the selected points and ***z****_k_* is the feature vector of the *k* – *th* point. ***C*** is the covariance matrix of the feature vector, *F*. The covariance matrix descriptor proposes a way to merge multiple features. Different choices of feature vectors are shown in Table 1, where *u* and *υ* are horizontal and vertical components of optical flow, *u_x_* and *υ_x_* are the first derivatives of horizontal and vertical optical flow in the *x* direction, respectively, *u_y_* and *υ_y_* are the first derivatives of the corresponding feature in the *y* direction, *u_xx_* and *υ_xx_* are the second derivatives in *x* direction and *u_yy_* and *υ_yy_* are the second derivatives in *y* direction. The flowchart of covariance matrix descriptor computation is shown in Figure 2. The optical flow and corresponding partial derivative characterize the inter-frame information or can be regarded as the movement information.

If proper parameters are given, classical kernels, such as Gaussian, polynomial and sigmoidal kernels, have similar performances [44]. In our work, the Gaussian kernel $\kappa \left(x_{i},x_{j}\right)=\exp\left(-\frac{∥x_{i}-x_{j}{∥}^{2}}{2\sigma^{2}}\right)$ is used. The covariance matrix is an element in the Lie group; the Gaussian kernel in Euclidean spaces is not suitable. The Gaussian kernel in the Lie group is defined as [45,46]:

(45) $$\kappa (X_{i},X_{j})=\exp(-\frac{\left\|\log\left(X_{i}^{-1}X_{j}\right)\right\|}{2\sigma^{2}}),(X_{i},X_{j})\in G\times G$$

where ***X**_i_* and ***X**_j_* are matrices in Lie group *G*; the parameter *σ* determines the scale at which the data is probed.

## 5. Abnormal Event Detection

In an abnormal event detection problem, it is assumed that a set of training frames, {*I*_1_*, I*_2_,…, *I_n_*} (the positive class), describing the normal behavior is obtained. The abnormal detection strategies relative to the online algorithms proposed in Section 3.3 and Section 3.4 are introduced below.

### 5.1. Online LS-OC-SVM Strategy

**Figure 3.**
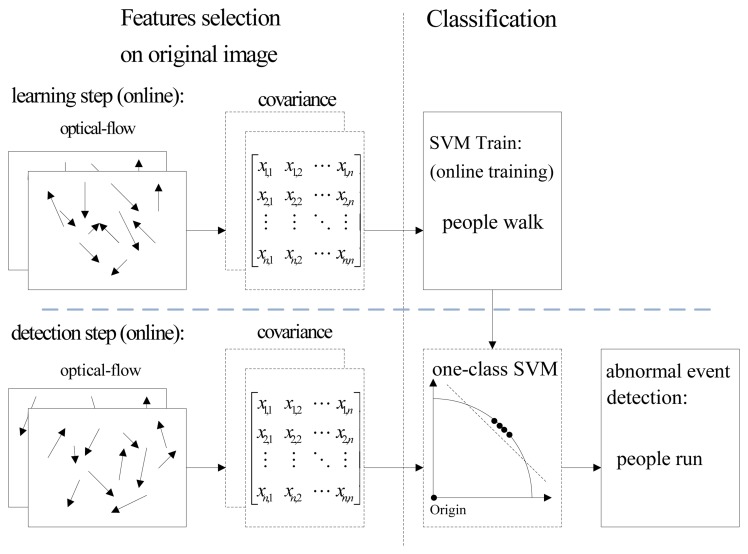
Major processing states of the proposed abnormal frame event detection method. The covariance of the *frame* is computed.

The general architecture of the abnormal event detection method via online least squares one-class SVM (online LS-OC-SVM) proposed in Section 3.3 is summarized in Algorithm 1; the flowchart is shown in Figure 3 and explained below.



**Algorithm 1**: Visual abnormal event detection via online least squares one-class support vector machine (LS-OC-SVM) and sparse online LS-OC-SVM.
**Require***n* training frames 
${\left\{I_{i}\right\}}_{i=1}^{n}$and the corresponding optical flow 
${\left\{OP_{i}\right\}}_{i=1}^{n}$.Compute the covariance matrix of each frame.
(46)$$\begin{array}{ccc} \left\{OP_{1},OP_{2},\ldots ,OP_{n}\right\} & \to & \left\{C_{1},C_{2},\ldots ,C_{n}\right\} \end{array}$$
(a)*Online strategy:* Applying LS-OC-SVM on the small subset of training samples to calculate the coefficient matrix.
(47)$\left\{C_{1},C_{2},\ldots ,C_{m}\right\},1\leq m\ll n\overset{online}{\to }\text{coefficient matrix}\;\left[K\right]{\left[\alpha -\rho \right]}^{⊤}$(b)*Sparse online strategy:* Applying LS-OC-SVM to train the initial dictionary, *C*_
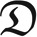
_, offline.
(48)$C_{D}=\left\{C_{1},C_{2},\ldots ,C_{m}\right\},1\leq m\ll n\overset{\text{offline}}{\to }\text{coefficient matrix}\;\left[K\right]{\left[\beta -\rho \right]}^{⊤}$(a)*Online strategy:* Applying online LS-OC-SVM on the remaining samples to calculate the coefficient matrix.
(49)$\left\{C_{m+1},C_{m+2},\ldots ,C_{n}\right\},\left[K\right]\overset{\text{online}}{\to }\text{coefficient matrix}\;\left[K\right]{\left[\alpha -\rho \right]}^{⊤}$(b)*Sparse online strategy:* Applying sparse online LS-OC-SVM on the remaining samples to calculate the coefficient matrix and to update the dictionary.
(50)$$\begin{array}{l} \left\{C_{D},C_{K}\right\},m<k\leq n\overset{\text{sparse online}}{\to }\text{coefficient matrix}\;{\left[\beta -\rho \right]}^{⊤}\\ \{\begin{array}{lll} C_{D}:=C_{D}\cup C_{k}, & \text{if} & \epsilon_{t}\geq \mu_{0}\\ C_{D}:=C_{D}, & \text{if} & \epsilon_{t}<\mu_{0} \end{array} \end{array}$$Each frame ***C****_n_*_+l_ is classified via LS-OC-SVM.

**Step 1:** The first step consists of calculating the covariance matrix descriptor of the training frames. The features could be chosen as any form shown in Table 1. This step can be generalized as:
  (51) $$\left\{OP_{1},OP_{2},\ldots ,OP_{n}\right\}\to \left\{C_{1},C_{2},\ldots ,C_{n}\right\}$$
  where {*OP*_1_,*OP*_2_,…, *OP_n_*} are the image optical flows of the 1*st* to *n* – *th* frames; {***C***_1_,***C***_2_,…, ***C****_n_*} are the covariance matrix descriptors.
**Step 2:** The second step is applying LS-OC-SVM on a small subset of the training samples to calculate the coefficient parameters, ***α*** and *ρ*, in Equation (11). Consider a subset ${\left\{C_{i}\right\}}_{i=1}^{m}$, 1 ≤ *m* ≪ *n* of data selected from the training set ${\left\{C_{i}\right\}}_{i=1}^{n}$. Without loss of generality, assume that the first *m* frames are chosen. These *m* samples are trained offline. This step can be described in the following equation:
  (52) $$\left\{C_{1},C_{2},\ldots ,C_{m}\right\},1\leq m\ll n\overset{\text{offline}}{\to }\text{coefficient matrix}\;\left[K\right]{\left[\alpha -\rho \right]}^{⊤}$$
  where [***K*** ] and [***α*** − *ρ* ]^T^ are defined in Equation (11).
**Step 3:** After learning the first *m* samples, the coefficient matrices, ***K*** and [***α*** − *ρ* ]^T^, are obtained. The online LS-OC-SVM method (Section 3.3) is applied to learn the remaining *n* – *m* samples {***C****_m_*_+1_,***C****_m_*_+2_*…C_n_*}. This step can be expressed as:
  (53) $$\left\{C_{m+1},C_{m+2},\ldots ,C_{n}\right\},\left[K\right]\overset{\text{online}}{\to }\text{coefficient matrix}\;\left[K\right]{\left[\alpha -\rho \right]}^{⊤}$$
**Step 4:** Based on the coefficient matrix, [***α*** − *ρ* ]^T^, the distance of the training samples ${\left\{C_{i}\right\}}_{i=1}^{n}$ and the incoming test sample, ***C****_n_*_+_*_l_*, with respect to the decision plane is computed. By comparing the distances of the samples, an abnormal event is detected:
  (54) $$dis\left(C_{n+l}\right)=\frac{\left|\left(\sum_{i=1}^{n}\alpha_{i}\kappa (C,C_{i})-\rho \right)\right|}{\left\|\alpha \right\|}$$
  (55) $$=\{\begin{array}{ccc} 1 & \text{if} & f(C_{n+l})\geq T_{dis}\\ -1 & \text{if} & f(C_{n+l})<T_{dis} \end{array}$$

where ***C****_n_*_+_*_l_* is the covariance matrix descriptor of the (*n* + *l*) – *th* frame needed to be classified, and *C_i_* is the sample of the training data. “1” corresponds to an abnormal frame; “ − 1” corresponds to a normal frame. *T_dis_* is the threshold of the distance, it is the maximum distance of the training samples to the hyperplane.

### 5.2. Sparse Online LS-OC-SVM Strategy

The abnormal event detection via sparse online least squares one-class SVM (sparse online LS-OC-SVM) is introduced below. A subset of the samples is chosen to form the dictionary, ***C***_
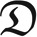
_, making a sparse representation of the training data. The initial dictionary, ***C***_
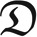
_, is learned offline. Each remaining training sample is learned one-by-one online. Meanwhile, it is checked to be included, or not, into the dictionary. The test datum is classified based on the dictionary. The feature extraction step (Step 1) and the detection step (Step 4) are the same as the ones presented in Section 5.1. Owing to the dictionary, the training steps are different.

**Step 2-sparse:** The second step is applying LS-OC-SVM to train the initial dictionary offline. The first *m* samples are the initial dictionary denoted as ***C***_
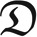
_. This step can be generalized as:

(56) $$C_{D}=\left\{C_{1},C_{2},\ldots ,C_{m}\right\},1\leq m\ll n\overset{\text{offline}}{\to }\text{coefficient matrix}\;\left[K\right]{\left[\beta -\rho \right]}^{⊤}$$

**Step 3-sparse:** After learning the initial dictionary, ***C***_
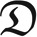
_, including the first *m*(1 ≤ *m* ≪*n*) samples, the remaining training samples, {***C****_m_*_+1_, ***C****_m_*_+2_, …, ***C****_n_*}, are learned via sparse online LS-OC-SVM described in Section 3.4. This step can be described in the following equations:

(57) $$\begin{array}{l} \left\{C_{D},C_{k}\right\},m<k\leq n\overset{\text{sparse online}}{\to }\text{coefficient matrix}\;{\left[\beta -\rho \right]}^{⊤}\\ \{\begin{array}{lll} C_{D}:=C_{D}\cup C_{k}, & \text{if} & \epsilon_{t}\geq \mu_{0}\\ C_{D}:=C_{D}, & \text{if} & \epsilon_{t}<\mu_{0} \end{array} \end{array}$$

where ***C***_
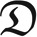
_ is the dictionary and ***C****_k_* is a new incoming remaining sample in the training dataset. According to the coherence criterion introduced in Section 3.4, if the new sample, ***C****_k_*, satisfies the dictionary updated condition, it will be included into the dictionary, ***C***_
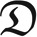
_.

## 6. Abnormal Event Detection Results

This section presents the results of experiments conducted to illustrate the performance of the two proposed classification algorithms, online least square one-class SVM (online LS-OC-SVM) and sparse online least square one-class SVM (sparse online LS-OC-SVM). The two-dimensional synthetic distribution dataset and the University of Minnesota (UMN) [47] dataset are used.

### 6.1. Synthetic Dataset via Online LS-OC-SVM and Sparse Online LS-OC-SVM

**Figure 4.**
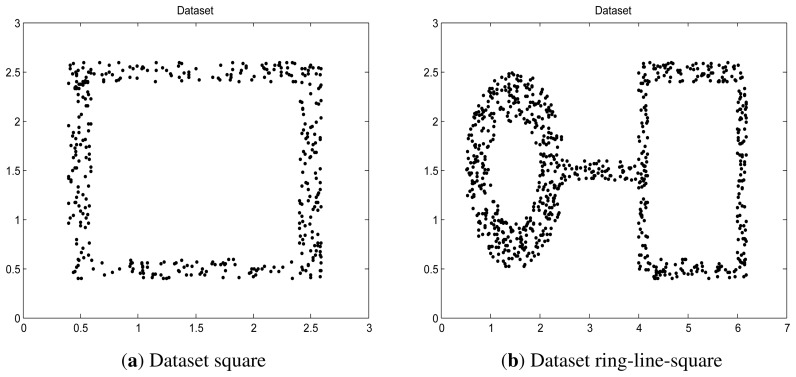
Synthetic dataset. (**a**) Square; (**b**) ring-line-square.

Two synthetic data, “square” and “ring-line-square” [48], are used. The “square” consists of four lines, 2.2 in length and 0.2 in width. In the area of these lines, 400 points were randomly dispersed with a uniform distribution. The “ring-line-square” distribution is composed of three parts: a ring with an inner diameter of 1.0 and an outer diameter of 2.0, a line of 1.6 in length and 0.2 in width, and a square the same as dataset “square” introduced above. 850 points are randomly dispersed with a uniform distribution. These two data are shown in Figure 4.

The first sample is used for initializing the online LS-OC-SVM proposed in Section 3.3; the 399 remaining samples in “square” and 849 remaining samples in “ring-ling-square” are learned in the online manner.

Via the sparse online LS-OC-SVM method proposed in Section 3.4, the first sample is trained offline, and this sample is considered the initial dictionary. Then, each arrival sample in 399 remaining samples in “square” and 849 remaining samples in “ring-ling-square” are checked by the coherence criterion to determine whether the dictionary should be retained or updated by including the new element.

**Figure 5.**
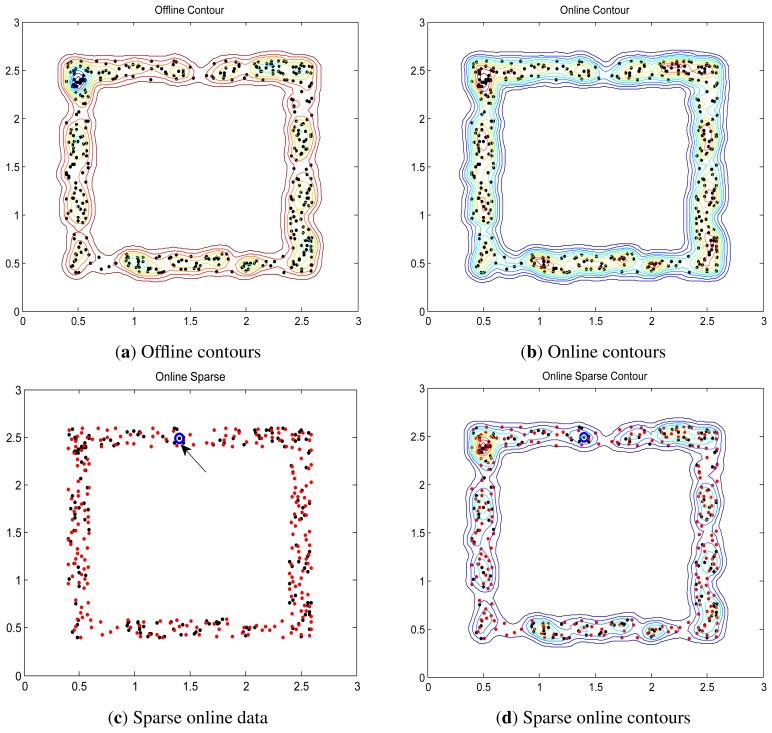
Offline, online LS-OC-SVM and sparse online LS-OC-SVM results of “square”. The figures might be viewed better electronically, in color and enlarged, (**a**) The contours when all the training data are learned as one batch offline; (**b**) The contours when the training data are learned via online LS-OC-SVM; (**c**) The blue circle (pointed out by the arrow) shows the original dictionary. The red points show the *232* new data included into the dictionary via sparse online LS-OC-SVM; (**d**) The contours when the training data are learned via sparse online LS-OC-SVM.

**Figure 6.**
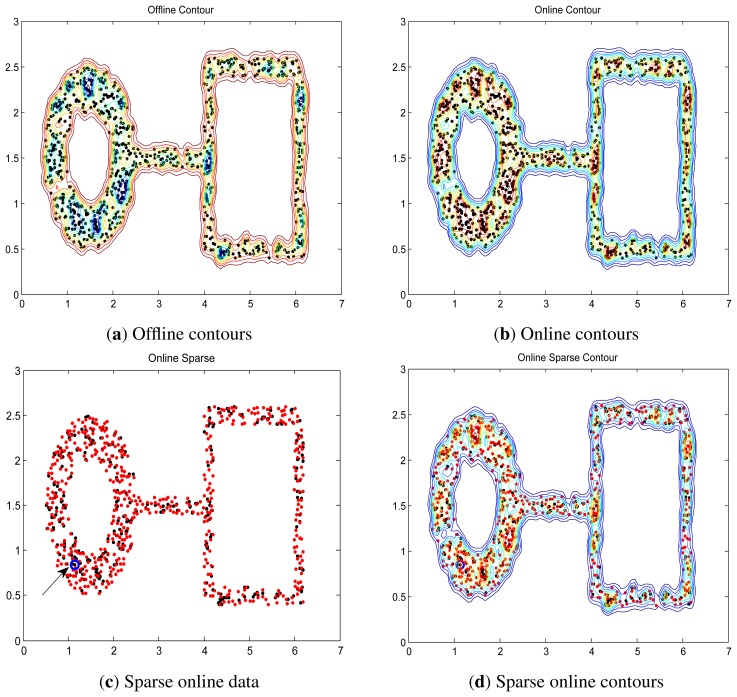
Offline, online LS-OC-SVM and sparse online LS-OC-SVM results of “ring-line-square”, (**a**) The contours when all the training date are learned as one batch offline; (**b**) The contours when the training data are learned via online LS-OC-SVM; (**c**) The blue circle (pointed out by the arrow) shows the original dictionary. The red points show the *534* new data included into the dictionary via sparse online LS-OC-SVM; (**d**) The contours when the training data are learned via sparse online LS-OC-SVM.

The distances are shown in contours illustrating the boundary. The contours of “square” and “ring-line-square” are shown in Figures 5 and 6, respectively. Gaussian kernel was used in these two data, with bandwidth *σ* = 0.065. The preset threshold of the coherence criterion is *μ*_0_ = 0.08. The detection results obtained by these two online training algorithms are the same as the ones when training data were learned in a batch model.

### 6.2. Abnormal Visual Event Detection via Online LS-OC-SVM

**Figure 7.**
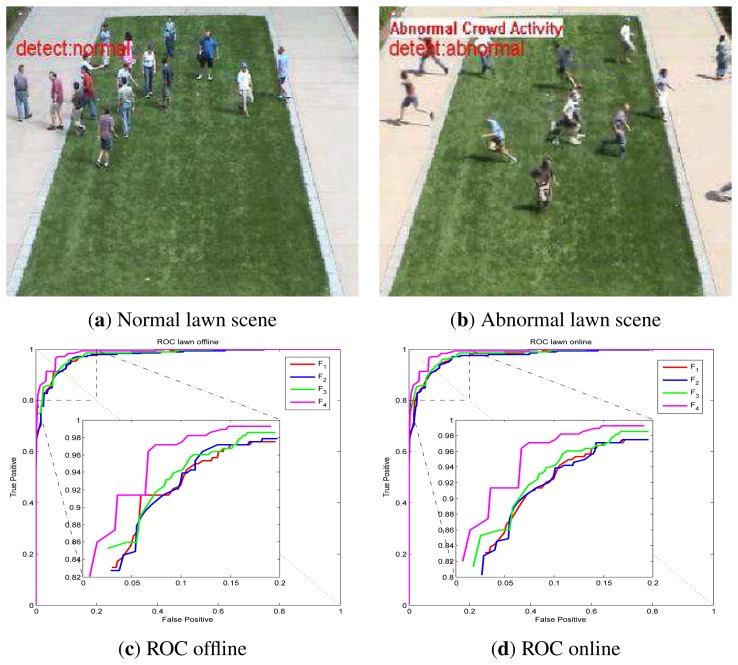
Detection results of the lawn scene, (**a**) The detection result of a normal frame; (**b**) The detection result of an abnormal panic frame; (**c**) Receiver operating characteristic (ROC) curve of covariance descriptors constructed from different features *F* of the lawn scene detection results via LS-OC-SVM. All the training samples are learned together offline. The biggest AUC value is 0.9874; (**d**) ROC curve of detection results via online LS-OC-SVM. The biggest AUC value is 0.9874.

**Figure 8.**
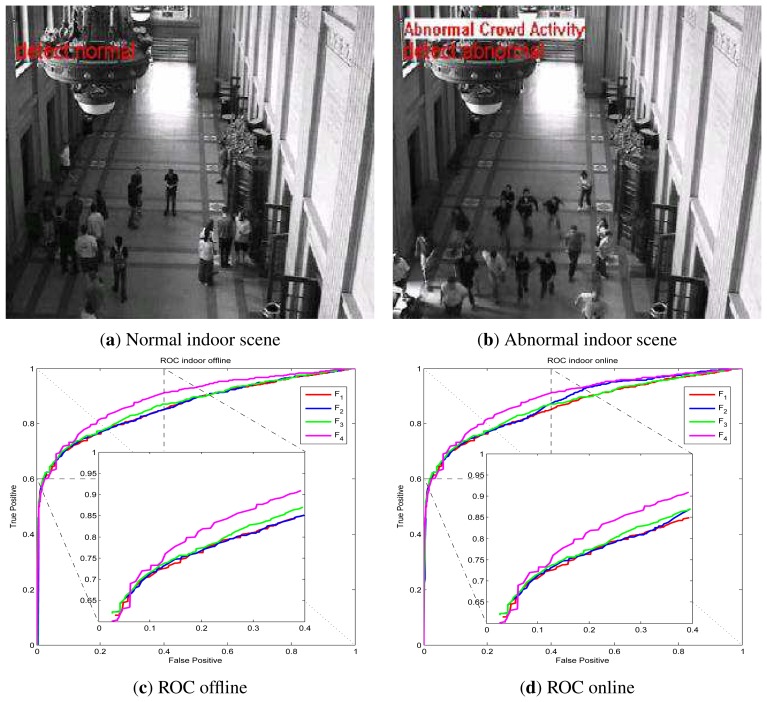
Detection results of the indoor scene. (**a**) The detection result of a normal frame; (**b**) The detection result of an abnormal panic frame; (**c**) ROC curve of covariance descriptors constructed from different features *F* of the indoor scene results via LS-OC-SVM. All the training samples are learned together offline. The biggest AUC value is 0.8900; (**d**) ROC curve of detection results via online LS-OC-SVM. The biggest AUC value is 0.8904.

**Figure 9.**
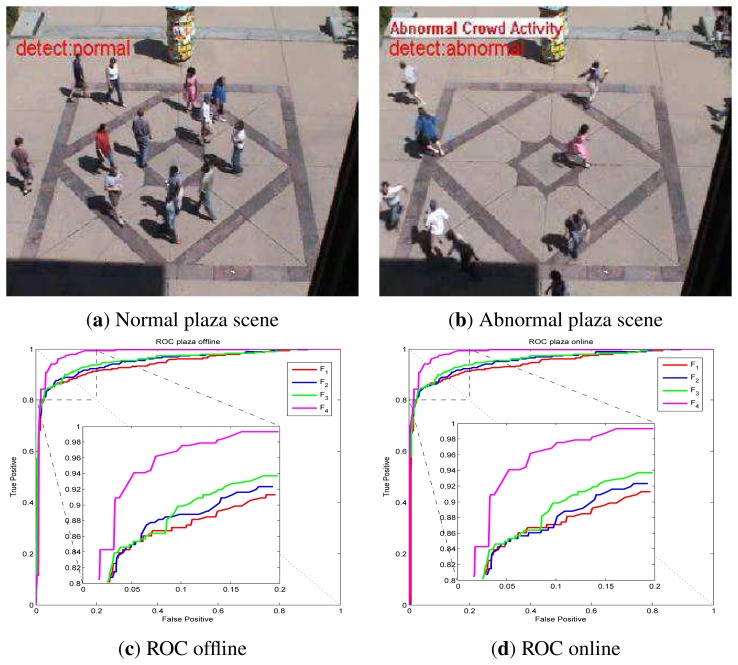
Detection results of the plaza scene. (**a**) The detection result of a normal frame; (**b**) The detection result of an abnormal panic frame; (**c**) ROC curve of covariance descriptors constructed from different features *F* of the plaza scene results via LS-OC-SVM. All the training samples are learned together offline. The biggest AUC value is 0.9800; (**d**) ROC curve of detection results via online LS-OC-SVM. The biggest AUC value is 0.9839.

UMN dataset detection results via online LS-OC-SVM proposed in Section 3.3 are shown below. The UMN dataset consists of eleven sequences of crowded panic escape events, which are recorded in a lawn, an indoor and a plaza scene. A frame where the people are walking in different directions is considered as a normal sample for training or for normal testing. A scene where the people are running is taken as an abnormal sample for testing. The detection results of the lawn scene, the indoor scene and the plaza scene are shown in Figures 7, Figure 8 and Figure 9, respectively. A Gaussian kernel for the covariance matrix in the Lie group is used. Various values of the variance, *σ*, in the Gaussian function and the penalty factor, *C*, are chosen to form the receiver operating characteristic (ROC) curve. In the indoor scene, time lags of the frame labels lead to the lower area under the ROC curve (AUC) value. In the last few frames, labeled as abnormal of abnormal sequences, there are no people, while, in the training samples, there are no people in the upper half of the image. The covariance of the training frame is similar to the covariance of the abnormal frame without people. Our covariance feature descriptor-based classification method cannot distinguish between these two situations. However, this issue can be resolved by utilizing the foreground information. For example, if there are no moving objects in the frame, this frame is immediately classified as abnormal. The results of these three scenes show that the covariance descriptor can distinguish between normal and abnormal events. The performance of online LS-OC-SVM is almost the same as that of the offline method.

### 6.3. Abnormal Visual Event Detection via Sparse Online LS-OC-SVM

UMN dataset abnormal event detection results via sparse online LS-OC-SVM proposed in Section 3.4 are presented. Taking the lawn scene as an example, the first normal covariance matrix descriptor from the training samples is included into the dictionary firstly; then, the remaining training covariance descriptors are learned online by the sparse online LS-OC-SVM method. The ROC curve of the detection results of the lawn scene, the indoor scene and the plaza scene are shown in Figure l0a–c, respectively.

The resulting performances when all training samples are learned offline via one-class SVM (OC-SVM), learned via least squares one-class SVM (LS-OC-SVM), learned via online least squares one-class SVM (online LS-OC-SVM) and learned via sparse online least squares one-class SVM (sparse LS-OC-SVM), are shown in Table 2. The LS-OC-SVM algorithm obtains better performance than the original OC-SVM. The performances of online and sparse online strategy results are similar to the resulting performances when all training samples are learned offline. The sparse online strategy can be computed efficiently and can adapt to the memory requirement.

The resulting performances of the covariance matrix descriptor-based online least squares one-class SVM method, and of state-of-the-art methods, are shown in Table 3. The covariance matrix-based online abnormal frame detection method obtains competitive performance. In generally, our sparse online LS-OC-SVM method is better than others, except sparse reconstruction cost (SRC) [49]. In that paper, multi-scale histogram of optical flow (HOF) was taken as a feature and a testing sample was classified by its sparse reconstruction cost, through a weighted linear reconstruction of the over-complete normal basis set. However, the computation of the HOF takes more time than the computation of covariance. By adopting the integral image [43], the covariance matrix descriptor of the subimage can be computed conveniently. The covariance descriptor can appropriately be used to analyze partial image movement. In [49], the whole training dataset was saved in the memory in advance; then, the dictionary was chosen as an optimal subset for reconstructing. Our sparse online LS-OC-SVM strategy enables one to train the classifier with sequential inputs. This property makes our proposed method extremely suitable to handle large volumes of training data, while the method in [49] fails to work due to lack of memory.

## 7. Conclusions

In this paper, we proposed a method to detect abnormal events via online least squares one-class SVM (online LS-OC-SVM) and sparse online least squares one-class SVM (sparse online LS-OC-SVM). Online LS-OC-SVM learns training samples sequentially; sparse online LS-OC-SVM incorporates the coherence criterion to form the dictionary for a sparse representation of the detector. The covariance matrix descriptor encodes the movement feature of the frame to distinguish between normal and abnormal events. The proposed detection algorithms have been tested on a synthetic dataset and a real-world video dataset yielding successful results in detecting abnormal events.

## Figures and Tables

**Figure 10.**
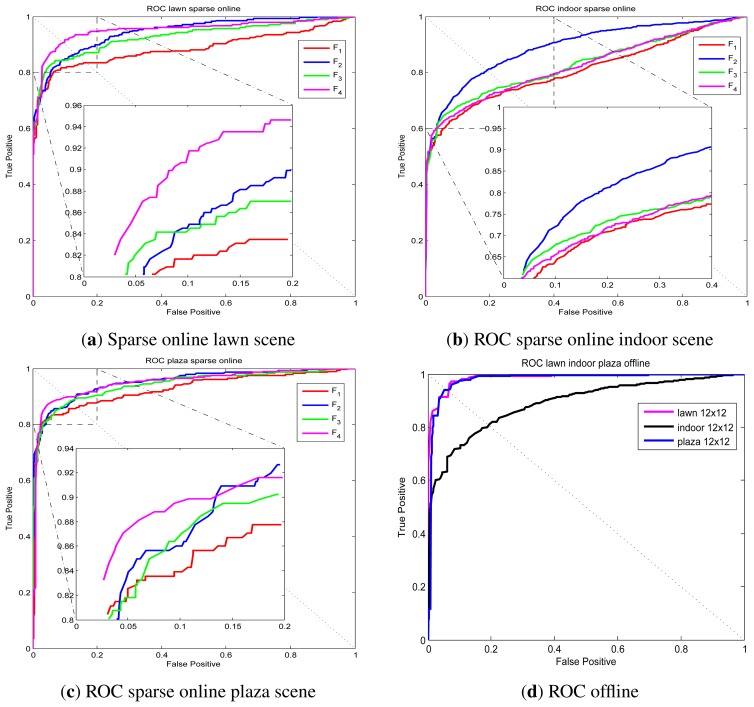
ROC curve of University of Minnesota (UMN) dataset. (**a**) Sparse online LS-OC-SVM results in the lawn scene. The biggest AUC value is 0.9510; (**b**) Sparse online LS-OC-SVM results in the indoor scene. The biggest AUC value is 0.8886; (**c**) Sparse online LS-OC-SVM results in the plaza scene. The biggest AUC value is 0.9515; (**d**) The ROC curve of the best performance of the lawn, plaza and indoor scene when the training samples are learned via LS-OC-SVM offline. The biggest AUC values of the lawn, indoor and plaza scene are 0.9874, 0.8900 and 0.9800.

**Table 1.** Different choices of feature *F* to construct the covariance descriptor.

	**Feature Vector*F***
*F*_1_(6 × 6)	[*y x uυu_x_u_y_* ]
*F*_2_(6 × 6)	[*yxuυ υ_x_υ_y_* ]
*F*_3_(8 × 8)	[*y xuυu_x_u_y_υ_x_υ_y_* ]
*F*_4_(12 × 12)	[*y xuυu_x_u_y_υ_x_υ_y_u_xx_u_yy_υ_xx_υ_yy_* ]

**Table 2.** AUC of the abnormal event detection method based on covariance descriptors constructed by different features *F* via OC-SVM (Section 3.1), original LS-OC-SVM learning training samples together offline (Section 3.2), online LS-OC-SVM (Section 3.3) and sparse online LS -OC-SVM (Section 3.4). The biggest value of each method is shown in bold.

Features	Area under ROC		
	lawn	indoor	plaza
offline OC-SVM			
*F*_1_(6 × 6)	0.9474	0.8381	0.9148
*F*_2_(6 × 6)	0.9583	0.8410	0.9192
*F_3_(8* × 8)	0.9656	0.8483	0.9367
*F*_4_(12 × 12)	**0.9798**	**0.8744**	**0.9782**
offline LS-OC-SVM			
*F*_1_(6 × 6)	0.9755	0.8605	0.9422
*F*_2_(6 × 6)	0.9738	0.8603	0.9489
*F*_3_(8 × 8)	0.9788	0.8662	0.9538
*F*_4_(12 × 12)	**0.9874**	**0.8900**	**0.9800**
Online LS-OC-SVM			
*F*_1_(6 × 6)	0.9755	0.8616	0.9403
*F*_2_(6 × 6)	0.9720	0.8730	0.9517
*F*_3_(8 × 8)	0.9795	0.8670	0.9563
*F*_4_(12 × 12)	**0.9874**	**0.8904**	**0.9839**
Sparse Online LS-OC-SV			M
*F*_1_(6 × 6)	0.8840	0.8077	0.9245
*F*_2_(6 × 6)	0.9435	**0.8886**	**0.9515**
*F_3_(8* × 8)	0.9269	0.8266	0.9428
*F*_4_(12 × 12)	**0.9510**	0.8223	0.9501

**Table 3.** The comparison of our proposed method with state-of-the-art methods for abnormal event detection in the UMN dataset. NN, nearest neighbor. SRC, sparse reconstruction cost. STCOG, spatial-temporal co-occurrence Gaussian mixture models.

Method	Area under ROC		
	lawn	indoor	plaza
Social Force [50]	0.96		
Optical Flow [50]	0.84		
NN [49]	0.93		
SRC [49]	0.995	0.975	0.964
STCOG [51]	0.9362	0.7759	0.9661
LS-SVM (Ours)	0.9874	0.8900	0.9800
Online (Ours)	0.9874	0.8904	0.9839
Sparse Online(Ours)	0.9510	0.8886	0.9515
